# Exploring dementia and neuronal ceroid lipofuscinosis genes in 100 FTD-like patients from 6 towns and rural villages on the Adriatic Sea cost of Apulia

**DOI:** 10.1038/s41598-021-85494-x

**Published:** 2021-03-18

**Authors:** Celeste Sassi, Rosa Capozzo, Monia Hammer, Chiara Zecca, Monica Federoff, Cornelis Blauwendraat, Nick Bernstein, Jinhui Ding, J. Raphael Gibbs, Timothy Price, Andrew Singleton, Giancarlo Logroscino

**Affiliations:** 1 Laboratory of Neurogenetics, National Institute on Aging, National Institutes of Health, Bethesda, MD USA; 2 Department of Clinical Neurology and Research, University of Bari, “Pia Fondazione Cardinale G. Panico”, Tricase, Lecce Italy; 3 Nurodegenerative Diseases Unit, Department of Basic Medical Science, Neuroscience and Sense Organs, University of Bari, Bari, Italy; 4 Charité – Universitätsmedizin Berlin, Charitéplatz 1, 10117 Berlin, Germany

**Keywords:** Clinical genetics, Mutation, Population genetics, Sequencing

## Abstract

Frontotemporal dementia (FTD) refers to a complex spectrum of clinically and genetically heterogeneous disorders. Although fully penetrant mutations in several genes have been identified and can explain the pathogenic mechanisms underlying a great portion of the Mendelian forms of the disease, still a significant number of families and sporadic cases remains genetically unsolved. We performed whole exome sequencing in 100 patients with a late-onset and heterogeneous FTD-like clinical phenotype from Apulia and screened mendelian dementia and neuronal ceroid lipofuscinosis genes. We identified a nonsense mutation in *SORL1* VPS domain (p.R744X), in 2 siblings displaying AD with severe language problems and primary progressive aphasia and a near splice-site mutation in *CLCN6* (p.S116P) segregating with an heterogeneous phenotype, ranging from behavioural FTD to FTD with memory onset and to the logopenic variant of primary progressive aphasia in one family. Moreover 2 sporadic cases with behavioural FTD carried heterozygous mutations in the *CSF1R* Tyrosin kinase flanking regions (p.E573K and p.R549H). By contrast, only a minority of patients carried pathogenic *C9orf72* repeat expansions (1%) and likely moderately pathogenic variants in *GRN* (p.C105Y, p.C389fs and p.C139R) (3%). In concert with recent studies, our findings support a common pathogenic mechanisms between FTD and neuronal ceroid lipofuscinosis and suggests that neuronal ceroid lipofuscinosis genes should be investigated also in dementia patients with predominant frontal symptoms and language impairments.

## Introduction

**Figure 1:**
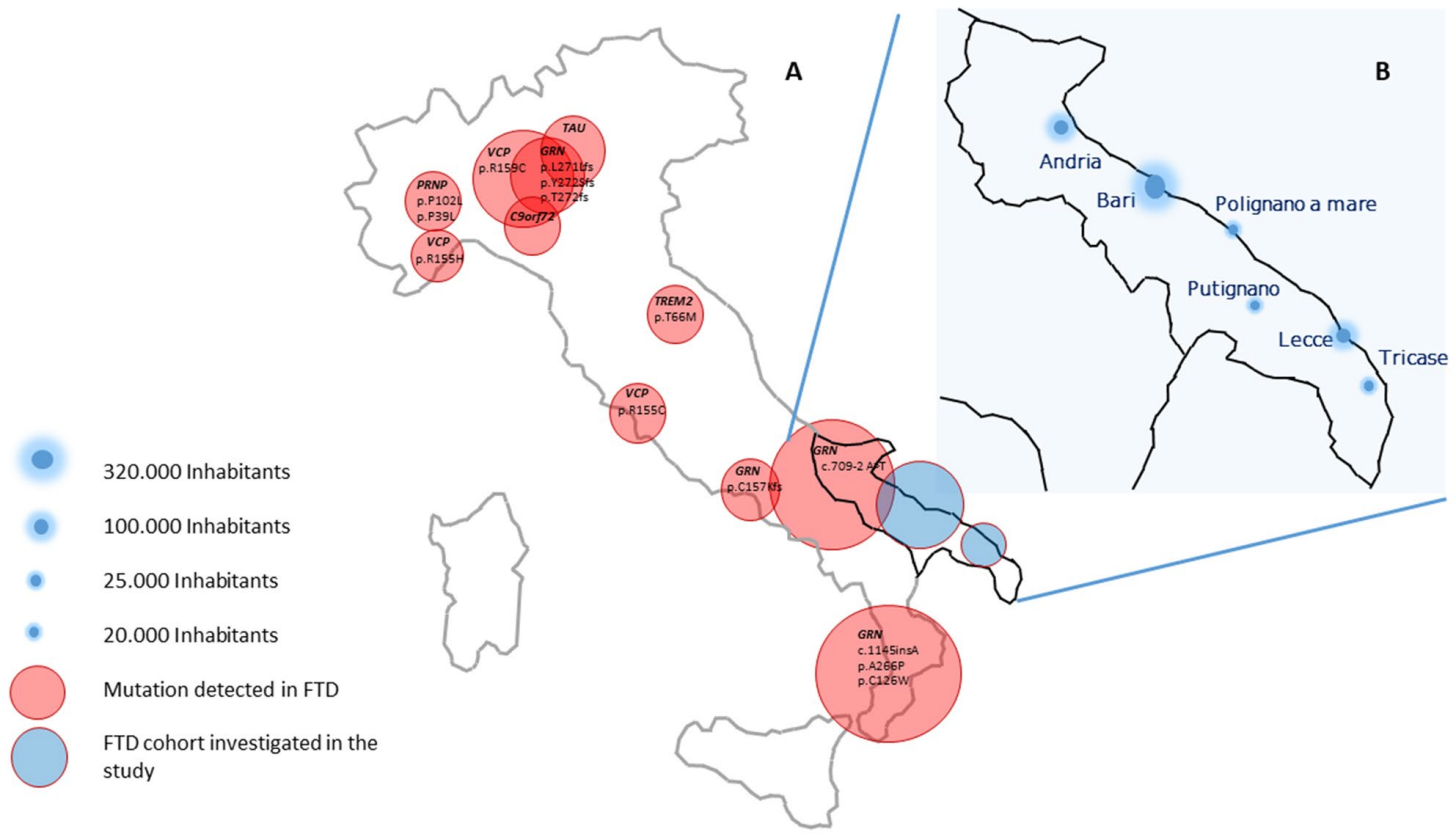
(**A**, **B**) Map of the genetic mutations in known FTD genes detected in Italy. Red circles describe the topographic area of the respective mutation. Blue circles display the area of provenience of the cohort investigated in the study, which has been shown in detail in Fig. 2B. Figure was generated using Power Point (https://www.microsoft.com/de-de/microsoft-365/powerpoint).

Frontotemporal dementia (FTD) refers to a clinical spectrum of disorders that are genetically, clinically, and neuropathologically heterogeneous. FTD is the second leading cause of early-onset dementia, after Alzheimer’s disease (AD)^1^. Genetics plays a pivotal role in the aetiology of FTD. 40–50% of FTD patients report a positive family history for disease^2^. Mutations in granulin *(GRN*) and microtubule-associated tau (*MAPT*) most typically cause early-onset (< 55 years) apparently Mendelian FTD. Hexanucleotide repeat expansions in the non-coding region of chromosome 9 open reading frame (*C9orf72*) underlie approximately 10% of all cases of FTD. Less frequently, mutations in the genes encoding TAR DNA-binding protein 43 (*TDP-43*)*,* valosin containing protein (*VCP*), and the charged multivesicular body protein 2B (*CHMP2B*), Ubiquilin 2 (*UBQLN2*), prion protein (*PRNP*) and Triggering Receptor Expressed on Myeloid Cells 2 (*TREM2*) have been reported^3–6^ and 16 pathogenic mutations in these genes have been detected in Italian FTD patients and explain part of the disease heritability (Table S1, Fig. 1).

Nevertheless, a large fraction of FTD families and apparently sporadic cases with late-onset disease does not carry mutations in these genes. On the other hand, a growing body of evidence pointed to likely shared pathogenic mechanisms between frontal dementing syndromes with extrapyramidal signs and neuronal ceroid lipofuscinosis (NCL)^7–10^. It is likely that very rare, Mendelian, coding and private mutations may explain some of the remaining genetic component of FTD. Given the rare frequency of the pathogenic variants and the lack of large multigenerational pedigrees, GWA and linkage studies are unlikely to effectively detect such variants. By contrast, whole exome sequencing (WES) is a powerful tool to investigate the genetic landscape underlying complex syndromes^11–13^. Thus, we have applied WES to screen Mendelian dementia and neuronal ceroid lipofuscinosis genes in 100 familial and apparently sporadic patients from 6 small and isolated towns and villages in Apulia displaying a heterogeneous FTD-like phenotype.

## Materials and methods

### Cohort

One hundred clinically diagnosed FTD patients (47% female; 16% familial, 84% sporadic) were recruited in 6 small and isolated towns and rural villages on the Adriatic Sea cost of Apulia, an Italian southern region characterized by a distinctive historic and geographic isolation over the centuries: Bari (320.000 inhabitants, 116 km^2^), Andria (99.671 inhabitants, 407 km^2^), Lecce (95.269 inhabitants, 238 km^2^), Putignano (26.000 inhabitants, 99,11 km^2^), Polignano a mare (17.925 inhabitants, 62 km^2^), Tricase (17.421 inhabitants, 43,64 km^2^) (Fig. 1). Given their isolation, these areas display a high level of consanguinity and parental isonomy^14^ and the local population is highly inbred and enriched for rare causative alleles or highly penetrant risk factors of strong effect size. The patients were selected from the SLAP-DEM registry for rare neurodegenerative disorders in Puglia, south Italy. 75% displayed behavioural FTD (bvFTD), 21% primary progressive aphasia (PPA), and 1% patient showed FTD-ALS. Three patients were diagnosed with AD, and 2 of these were then diagnosed with FTD with memory onset and PPA during the disease progression. Average age at onset was 63 years (43–85y), 55% of the patients presented early-onset (< 65 years) and 24% very early-onset (≤ 55 years) (Table 1). All patients were evaluated with a complete neuropsychological assessment, structural and functional neuroimaging, blood chemistry tests and electromyography if the neurological examination showed motor-neuronal signs.

**Table 1:** Cohort description.

Patients		Female (%)	AAO	bvFTD	bvFTD_ALS	PPA	AD	FTD with memory onset	Parkinsonism/extrapyramidal signs	Pyramidal signs	Diffuse atrophy	Frontotemporal atrophy	Frontal/frontotemporal hypoperfusion
Familial	16	9 (56.2)	64.8 (49–73)	11	0	2	1	2	0	5	6	9	2
Sporadic	84	38 (45.2)	62.1 (43–79)	66	1	17	0	0	6	0	7	22	8
Total	100	47 (47)		77	1	19	1	2	6	5	13	31	10

*AAO* age at onset, *bvFTD* behavioural frontotemporal dementia, *ALS* amyotrophic lateral sclerosis, *PPA* progressive primary aphasia.

This study and all experimental protocols were approved by the ethics committee on human research of the University Hospital and Polyclinic of Bari and the ethics committee of the Hospital of Lecce. Written informed consent was obtained from each subject enrolled in the study.

### DNA extraction

DNA was extracted from blood using the automated DNA extractor AutoGenFlex STAR (AutoGen, Holliston, MA, USA) according to the manufacturer’s protocol.

### *C9orf72* repeat expansion study

A repeat-primed PCR was performed to screen for the presence of the GGGGCC hexanucleotide repeat expansion in *C9orf72* as previously described^15^. Positive and negative controls were added to the polymerase chain reaction plate to assure accurate repeat analysis. Fragment length analysis was performed on an ABI 3730xl genetic analyzer (Applied Biosystems, Foster City, CA, USA) and data were analyzed using GeneScan software (version 4, ABI).

### Exome sequencing

In an attempt to rapidly identify the underlying genetic mutation/s, we performed whole-exome sequencing on the DNA of each of the 16 affected individuals belonging to different families and 84 apparently sporadic FTD patients. Whole-exome sequencing (WES) was performed using the Extended Nextera Rapid-Capture Exome kit (Illumina, San Diego, CA, USA) and the Illumina HiSeq 2000 System (Illumina, San Diego, CA, USA). Quality control (QC), alignment, preprocessing and subsequent variant discovery were performed in accordance with the genome analysis toolkit (GATK) best practices^16^. A mean QC score for each sample’s FASTQ sequence file by cycle was calculated to ensure the technical quality. Samples were aligned to hg19 reference genome with BWA^17^ and Picard (http://picard.sourceforge.net) calculated alignment metrics. During preprocessing, duplicate reads were marked and consequently ignored. Mappings around indels were locally realigned to correct mapping artifacts. Base quality scores were recalibrated to prepare reads for variant discovery. HaplotypeCaller^18^ was performed per sample variant discovery on prepared reads. Overall, more than 200 million sequencing reads were produced for each sample, covering more than 12 billion bases. Approximately 98% of these were aligned to the human reference genome (hg19). On average, 92% of exome capture baits had at least 10 × depth and 87% at least 30 × depth.

The cohort was then jointly genotyped to capture the complete set of variants across all samples. Variant recalibration assigned a quality score commensurate to the probability of a SNP being a true variant. Then PLINK^19^ probed the heterozygosity, missingness and sex status of each sample. KING^20^ was used to measure pairwise relatedness between the subjects.

We used exome sequencing data to identify common (minor allele frequency [MAF] > 3%), rare (MAF < 3%), and very rare (MAF < 1%) coding variants in 26 genes causative for dementia (*GRN* [NM_002087], *MAPT* [NM_001123066], *VCP* [NM_007126], *C9orf72* [NM_001256054], *TREM2* [NM_001271821], *TYROBP* [NM_003332], *UBQLN2* [NM_013444], *PRNP* [NM_000311], *APP* [NM_000484], *PSEN1* [NM_000021], *PSEN2* [NM_000447], *SORL1* [NM_003105], *CSF1R* [NM_001288705], *NOTCH3* [NM_000435], *SNCA* [NM_001146055], *GBA* [NM_001171811] or Neuronal Ceroid Lipofuscinoses: *CLN10/CTSD* [NM_001909], *CLN1/PPT1* [NM_000391], *CLN3* [NM_001286105], *CLN5* [NM_006493], *CLN6* [NM_017882], *CLN7/MFSD8* [NM_152778], *CLN4* [NM_017882], *CLCN6* [NM_001256959], *CLCN7* [NM_001256959] and *SGSH* [NM_000199]. The coding variants detected in these genes have been collected and analysed (Table 2). The pedigrees of the families were drawn with Progeny (http://www.progenygenetics.com/).

**Table 2:** Coding mutations detected in the FTD-like cohort in Mendelian dementia and neuronal ceroid lipofuscinosis genes.

Gene	Pathaway	Position	Rs ID	cDNA	Aa change	Domain	ExAc	CADD	FTD carriers (tot = 100) (%)	CTRLS Carrier HEX (tot = 368)	Phenotype	AAO
*C9orf72*	Dementia										bvFTD	44
*GRN*	Dementia	chr17:42,427,084	Novel	c.G314A	p.C105Y		no	26.5	Bari_RA_bvFTD_48 Bari_RMA_bvFTD_51 (2%)	0	bvFTD bvFTD	57 60
*GRN*	Dementia	chr17:42,429,149	Novel	c.1165delT	p.C389fs		no	NA	RSA_bvFTD_50 (1%)	0	bvFTD	62
*GRN*	Dementia	chr17 :42,427,661	Reported	c.T415C	p.C139R		no	24	RSA_bvFTD_50 (1%)			
*MAPT*	Dementia	chr17: 44,067,289	Reported	c.C1228T	p.L410F		no	25.2	CM_bvFTD_60 (1%)	0	bvFTD	55
*VCP*	Dementia	chr9:35,062,983- 35,062,985	Novel	c.801_803del	p.267_268del		No	NA	Bari_CA_bvFTD_48 PPA_Mpd_01_42 (2%)	0	bvFTD PPA	59 64
*TREM2*	Dementia	chr6:41,126,423	Reported	c.C578A	p.P193Q		No	5.951	Bari_DP_PPA_35 (1%)	0	PPA	78
*PRNP*	Dementia	chr20:4,680,089- 4,680,112	Novel	c.223_246del	p.75_82del		No	NA	PPA_03_44 (1%)	0	PPA	66
*PSEN2*	Dementia	chr1:227,083,266	Novel	c.C1333G	p.Q445E		No	23.9	bvFTD_19_35 (1%)	0	bvFTD	69
*SORL1*	Dementia	chr11:121,421,343	Reported	c.C2230T	p.R744X	VPS10	No	38	H_II_2 H_II_4 (2%)	0	AD with severe language impairment/PPA	62 69
*SORL1*	Dementia	chr11:121,495,891	Reported	c.G6269T	p.G2090V		No	28.4	PPA_04_51 (1%)	0	PPA	55
*SORL1*	Dementia	chr11:121,440,881	NA	c.G3239A	p.R1080H		Benign 0.0116	23.1	FTD_AOS_01_59 (1%)	0	bvFTD	49
*CSF1R*	Dementia	chr5:149,441,322	rs376280561	c.G1717A	p.E573K	TK flanking region	0.0% Probably-dam	23.3	Bari_DFC_bvFTD_35 (1%)	0	bvFTD	78
*CSF1R*	Dementia	chr5:149,441,393	Reported	c.G1646A	p.R549H	TK flanking region	No	23.0	bvFTD_01_42 (1%)	0	bvFTD	64
*CTSD*	NCL	chr11:1,775,073	Reported	c.G1031A	p.G344D		No	22.6	bvFTD_10_38 (1%)	0	bvFTD	67
*PPT1*	NCL	chr1:40,555,177	Reported	c.132delT	p.F44fs		No	NA	bvFTD_03_34 (1%)	0	bvFTD	86
*CLCN6*	NCL	chr1:11,879,611	Reported	c.T346C	p.S116P		0.0233 Probably-dam	23.3	E_II_1 E_II_2 E_II_5 (3%)	0	FTD with memory onset PPA bvFTD	71 68 75
*SGSH*	NCL	chr17:78,184,307	Reported	c.G1453A	p.G485S		No	23.5	Bari_SA_bvFTD_57 RSA_bvFTD_50	0	bvFTD bvFTD	56 62

*Aa* amino-acid, *CTRLS* controls from HEX database^1^, *AAO* age-at onset.

### Variant filtering

All detected variants were functionally annotated with ANNOVAR^21^ and KGGSeq^22^. Variants were filtered for (1) heterozygous non synonymous, stop gain/loss, frameshift insertions/deletions and splice mutations that were (2) absent or very rare (minor allele frequency ≤ 0.001) in the public databases NHLBI ESP6500 (http://evs.gs.washington.edu/EVS/ ) and ExAC03 (http://exac.broadinstitute.org/) and (3) predicted pathogenic by at least one of the following in silico software algorithms: MetaSVM, MetaLR^23^ and CADD Phred score ≥ 20 (University of Washington and HudsonAlpha Institute for Biotechnology, Huntsville, AL).

### Sanger sequencing

To verify that the variants reported in this study were not an artifact of the exome sequencing process, Sanger sequencing was performed using an ABI BigDye Terminator Cycle Sequencing Kit on an ABI 3730xl Sequencer. Sequence traces were analyzed using Sequencher (version 4.2; Gene Codes Corporation, Ann Arbor, MI, USA).

The pipeline of our study has been described in Fig. S1.

All methods were carried out in accordance with relevant guidelines and regulations.

## Results

We identified 17 rare coding variants in the selected genes. Most of them, 12/17 (70%), were singletons, 5 were novel variants. In our cohort *TYROBP, UBQLN2, APP, PSEN1, NOTCH3, SNCA, GBA, CLN2, CLN3, CLN5* did not present any rare coding variant (Table 2).

### Dementia genes

Variants in *GRN* were detected in three subjects (3%). Two carried the same mutation (c.G314A, p.C105Y), previously shown to affect both the secretion of PGRN in cultured cells and the elastase cleavage of PGRN into GRN^24^. One patient (RSA_bvFTD_50) referred as apparently sporadic, carried two different variants in *GRN*. One missense mutation (c.T415C, p.C139R) in exon 5 leading to a predicted partial loss of functional protein and suggested as pathogenic by in silico and in vitro studies^25^. This mutation has been associated with behavioral frontotemporal dementia, semantic dementia, Alzheimer’s disease and corticobasal syndrome^26^. The second variant is a novel (e.g. not present in public databases) nucleotide deletion (c.1165delT, p.C389fs) in exon 10 predicted to give rise to a frameshift leading to the partial loss of function (Table 2). This patient presented with behavioral symptoms at age 63 (apathy, social retire and delusions). Four years later, he was completely socially inappropriate, unable to communicate and dependent in all daily activities with sphincter incontinence.

Only one individual (1%) carried a pathologic *C9orf72* hexanucleotide repeat expansion (37 repeats). The carrier was a male sporadic case and displayed bvFTD with non-fluent aphasia and a very early age at onset (44 years).

We report a rare and likely non-pathogenic variant in *PSEN2* p.Q445E, mapping outside the alpha helix surface of the transmembrane domains (TMs), where all the pathogenic mutations have been reported (alpha-helix rule)^27^.

Interestingly, we detected also 2 variants in *CSF1R* TK flanking regions (aa 538–581 and 911–972) (p.E573K and p.R549H). Although mutations in the TK domain (exons 12–22, aa 582–910) have been reported as pathogenic^12^, mutations in the TK flanking regions have been linked to AD^28^ and particularly p.E573K is characterized by a significantly decreased autophosphorylation compared to the wild-type CSF1R and has been previously reported in a patient presenting ischemic embolic stroke without the classical HDLS clinical feature but periventricular white matter abnormalities, unrelated to the recent infarct^29^.

**Figure 2:**
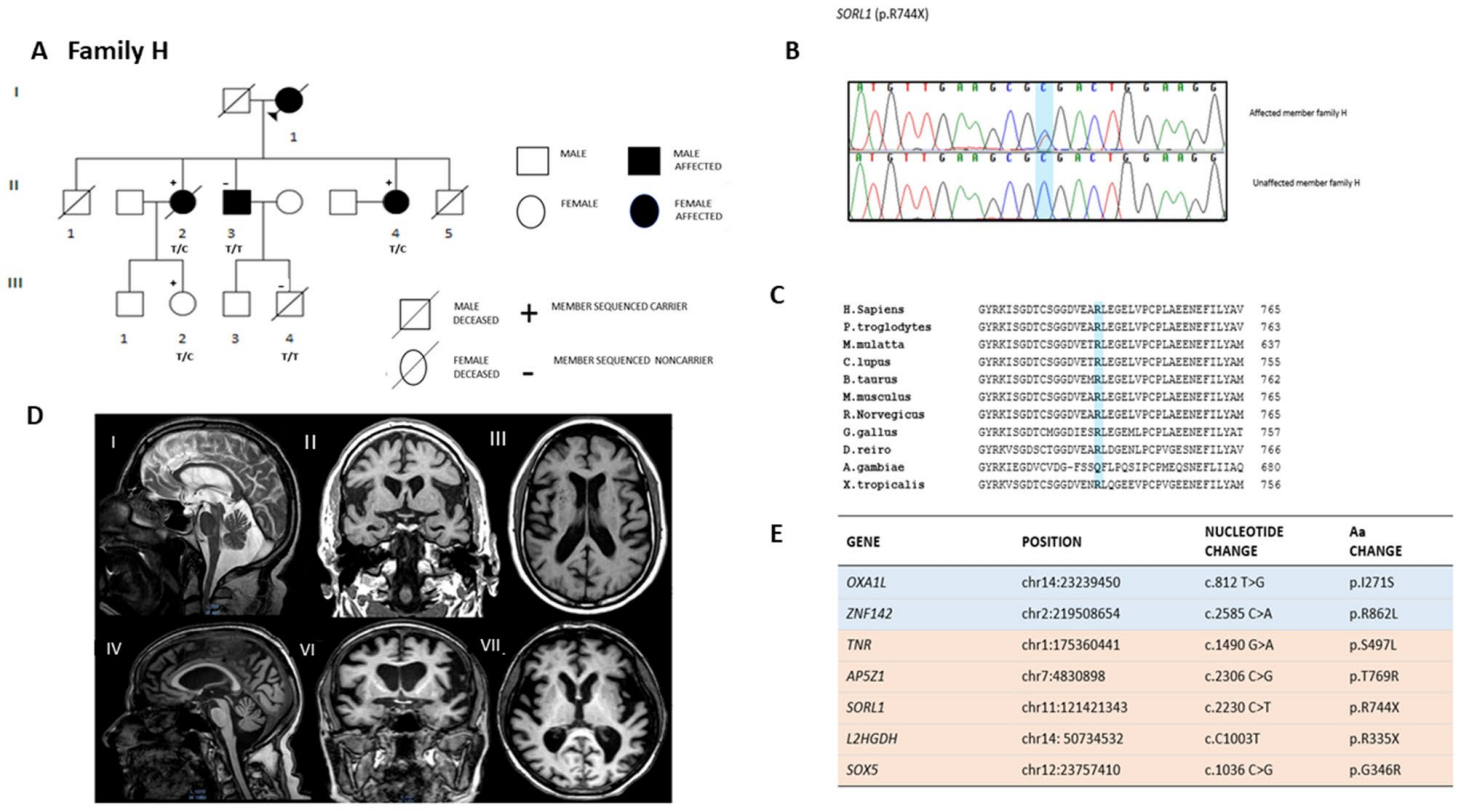
(**A**) Family H Pedigree. (**B**) *SORL1* p.R744X Sanger sequencing chromatogram in an affected family member and in a control. (**C**) Conservation of *SORL1* p.R744X across different species. (**D**) Brain MRI scans of 2 members of family H (HII3 and HII4). Sagittal (I, IV), coronal (II, VI), and axial (III, VII) T1-weighted images. (**E**) List of nonsynonymous coding variants segregating or detected in some of the affected members within Family H. In light blue are the variants which meet all the filter criteria: (1) novel variants; (2) segregating with the disease; (3) predicted as damaging by at least 2 out of 3 in silico prediction softwares (MUTATION TASTER, POLYPHEN2, SIFT) and 4) highly expressed in the brain and highly conserved (Grantham > 50, PhastCons > 0.4 and GERP > 4). In orange, variants which not fully segregate with the disease but may contribute to the disease phenotype. The pedigree of family H was drawn with Progeny (http://www.progenygenetics.com/).

Moreover, we report one *SORL1* mutation in the valosin-containing protein (VCP) domain (p.R744X) that was associated to AD with severe language impairment and PPA and was not detected in a member of the same family that had been initially diagnosed with AD and successively with FTD with memory onset (Table 3, Fig. 2A, B). *SORL1* p.R744X was also found in an unaffected family member from the third generation (HIII2), aged 42 years, who should be considered at risk (average age at onset in Family H is 68 years).

**Table 3:** Family H clinical features.

Family member	Genetic screening	Gender	AAO	AAD	Duration	First symptom	Memory deficit	Behavioral problem	Language problems	Neurologic evaluation	CT/MRI	Clinical diagnosis
HI1	Not performed	F	60y	65y	5y	Behavioral, personality and mood changes	NA	Behavioral, personality and mood changes	NA	NA	NA	Clinical history was referred by relatives. Probable FTD-like syndrome
HII1	Not performed	M	NA	70y, lung cancer	NA	NA	NA	NA	NA	NA	NA	NA
HII2	Negative	F	62y	80y	18y	Memory problems and disorientation	Yes	None known	Paraphasic errors, agrammatism, mutism	Spastic hypertony, increased DTR, aphasia, global cognitive impairment	*TC*: diffuse cerebral atrophy	AD with severe language impairment/PPA
HII3	Negative	F	73y	Alive	5y	Short-term memory deficits, disorientation	At onset, short-term memory impairment. Later, long-term memory problems, attention deficit	None known	None known	Right superior limb with II motoneuron signs. EMG negative for any pathological sign	*MRI*: predominant anterior atrophy	FTD with memory onset
HII4	Negative	F	69y	Alive	6y	Language problems	None known	None known	Alexia, agraphia, perseverative language, mutism	Motor aphasia	*MRI*: predominant anterior atrophy *SPECT*: left temporoparietal hypoperfusion	PPA
HII5	Not performed	M	NA	60y, myocardial infarction	NA	NA	NA	NA	NA	NA	NA	NA
HIII5	Not performed	M	NA	13y, road traffic accident	NA	NA	NA	NA	NA	NA	NA	NA

*AAO* age at onset, *AAD* age at death, *AD* Alzheimer’s disease, *PPA* primary progressive aphasia, *bvFTD* behavioral frontotemporal dementia, *MRI* magnetic resonance imaging, *SPECT* single photon emission computed tomography, *DTR* deep tendon reflex, *EMG* electromyography, *Y* years, *NA* not available, *F* female, *M* male.

The novel stop-gain mutation in *SORL1* (p.R744X) clusters in a very well conserved domain across different species (Fig. 2C) and maps to exon 16, carrying several mutations that have been linked to familial and sporadic AD^30–32^. None of the sporadic FTD cases carried *SORL1* variants in the VCP domain.

While a second heterozygous LoF mutation was identified within *L2HGDH* (p.R335X) in family H, this nonsense mutation did not segregate with disease. Finally, we report 4 mutations that cluster within genes highly expressed in the brain and already associated to developemental cognitive impairment and intellectual disability (*TNR* [p.S497L], *AP5Z1*[p.T769R]*, SOX5* [p.G346R], *ZNF142* [p.R862L])^33^ (https://www.omim.org/) (Table S3, Fig. 2E). Moreover, *OXA1L* has been linked to mitochondrial encephalopathy and *AP5Z1* and *SOX5* to hereditary spastic paraplegia and ALS, respectively (https://www.omim.org/) ^34^. Although these mutations do not meet all the filter criteria, given the critical role in CNS development, they may be disease modifiers.

It is possible that these mutations (*CLCN6* p.S116P, *SORL1* p.R744X, *L2HGDH* p.R335X) lead to haploinsufficiency due to a nonsense-mediated decay (NMD) or either the generation of a truncated protein. Due to the lack of RNA samples available, it was not possible to perform a transcript analysis and demonstrate the absence of the mutant allele and therefore discriminate between the two mechanisms.

### Family H

The clinical course of patients within Family H is characterized mainly by language impairment (HII2, HII4) and memory problems (HII2 and HII3) (Fig. 2A). The clinical diagnosis of affected family members includes probable AD, PPA and FTD with memory onset. The clinical features of the family members are summarized in Table 3.

The proband of the family died at 65 years of age and no samples were available for genetic evaluation. However, relatives described the patient as suffering from a dementing syndrome with behavioral and personality changes at the age of 60 years old.

#### HII2

At 62 years of age, the patient presented with memory impairment and spatiotemporal disorientation. Eight years after the onset of symptoms, she developed language problems that progressively worsened over four years with anomie, paraphasic errors and agrammatism progressing to mutism. At the age of 74 years, the patient was bed-ridden and completely dependent for all the daily activities. A neurological examination revealed spastic hypertony in all four limbs, increased and severe deep tendon reflex, mixed aphasia and global cognitive impairment. The clinical diagnosis was consistent with AD with severe language impairment. The patient deceased, aged 80 years old.

#### HII3

At 73 years of age, the patient developed short-term memory problems, depression and showed apathetic behavior. Three years later, aged 76 years, a neuropsychological examination revealed spatiotemporal disorientation. Long-term memory impairment and attention–execution deficits characterized the disease progression. An MRI scan, showed a marked anterior atrophy (Fig. 2D). The patient has been diagnosed with FTD with memory onset.

#### HII4

At 69 years of age, the patient presented with language impairment (anomie and stutter). Over the next four years, language problems progressed to complete mutism with alexia and agraphia. A neurological examination revealed a complete motor aphasia without any remarkable language comprehension impairment. Her behavior was socially appropriate. An MRI scan, performed three years after the onset of symptoms, revealed predominant anterior atrophy (Fig. 2D). A SPECT scan showed left temporo-parietal hypoperfusion. The patient was diagnosed with PPA.

Importantly, HII2 presented AD dementia and spastic paraplegia at the 4 limbs. Although this is a typical sign of patients with pathogenic mutations in *PSEN1*^35^, we have not detected any coding mutation in *PSEN1* in this family. However, we report a rare heterozygous missense mutation in *AP5Z1*, a gene that have been associated to autosomal recessive spastic paraplegia type 48 (SPG48)^36^. Nevertheless, the MRI did not present any typical feature of hereditary spastic tetraparesis: no periventricular white matter hyperintensities or thin corpus callosum (Fig. 2D). However, we cannot exclude that this mutation may modify the disease phenotype.

### Neuronal ceroid lipofuscinosis genes

**Figure 3:**
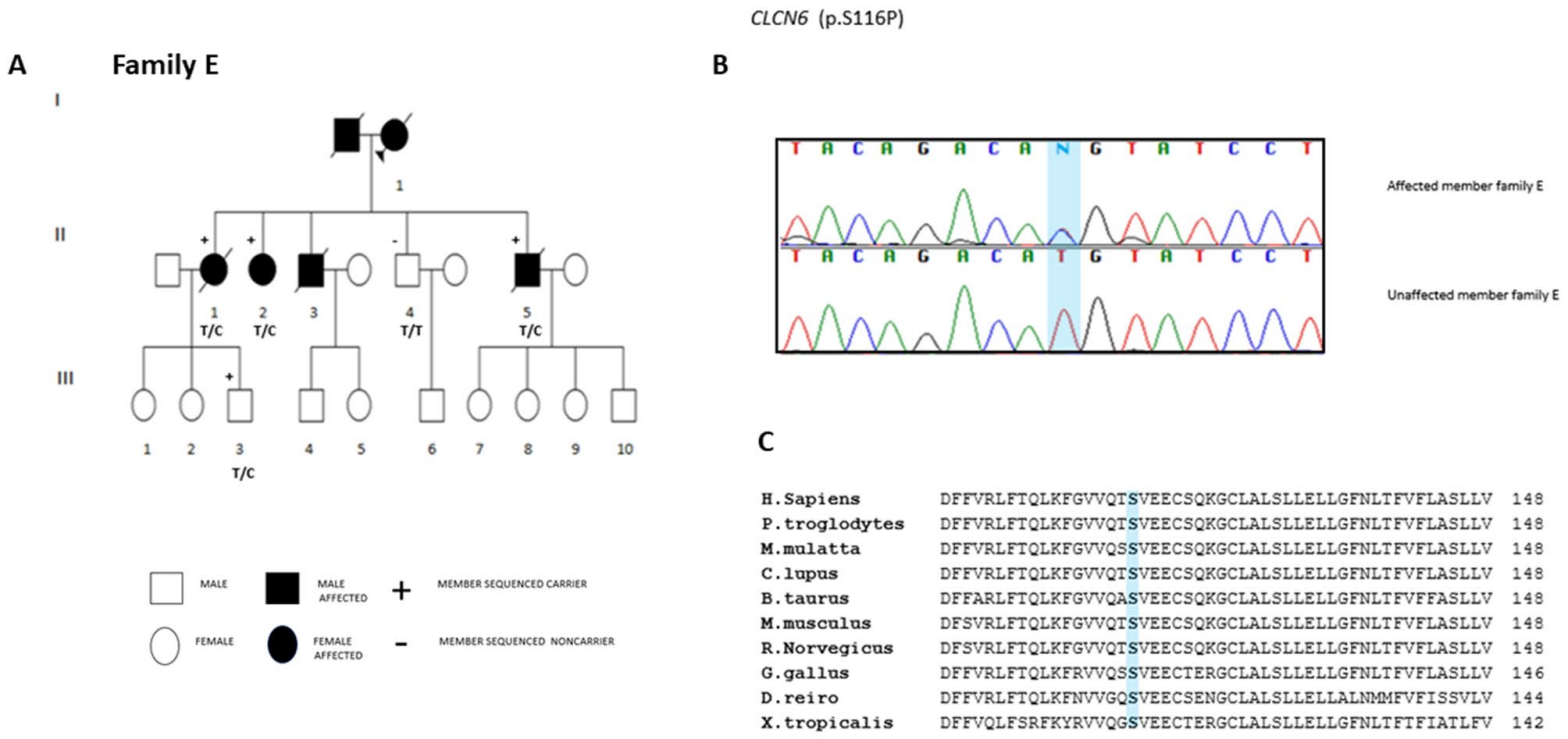
(**A**) Family E Pedigree. (**B**) *CLCN6* p.S116P Sanger sequencing validation. (**C**) Conservation of CLCN6 p.S116P across different species. The pedigree of family E was drawn with Progeny (http://www.progenygenetics.com/).

We report a novel and likely pathogenic variant identified in *CLCN6* (p.S116P) leading to a T to C transition in the last nucleotide of exon 5 (c.346 in coding DNA reference sequence NM_001286.2), at position − 1 of the exon 5 splice donor site (Fig. 3B). The same mutation may alternatively result in exon 5 skipping or act as a missense mutation (c.T346C, predicting a p.S116P substitution), that may modify the protein activity.

This heterozygous mutation (*CLCN6,* p.S116P) segregates with the FTD-like phenotype in Family E and has been found in all the three affected siblings of Family E (EII1, EII2, EII5). Furthermore, also an asymptomatic member in the third generation (EIII3) carried the *CLCN6* p.S116P variant. EIII3, aged 52 years, was likely too young to manifest the phenotype (average age at onset for the affected members was 71.3 years) (Table 4, Fig. 3A, B). Importantly, *CLCN6* p.S116P was the only novel putative loss of function mutation predicted as damaging by at least 2 out of 3 in silico prediction softwares (MUTATION TASTER, POLYPHEN2, SIFT), highly expressed in the brain and highly conserved (Grantham > 50, PhastCons > 0.4 and GERP > 4) (Table S4, Fig. 3C), segregating with the disease phenotype in Family E, therefore this was the most likely mutation that could have explained the disease in this family.

**Table 4:** Family E clinical features.

Family member	Genetic screening	Gender	AAO	AAD	Duration	First symptom	Memory deficit	Behavioral problem	Language problems	Neurologic evaluation	CT/MRI	Clinical diagnosis
EI1	Not performed	F	NA	65y, pneumonia	NA	Referred cognitive problems	Referred memory impairment	None known	None known	NA	NA	Clinical history was referred by relatives. Probable dementing syndrome
EII1	Negative	F	71y	80y, pneumonia	9y	Short-term memory problems, Attention deficit	Short-term memory problems, Attention deficit	Mild personality changes, later in the disease	None known	Disoriented	NA	FTD with memory onset
EII2	Negative	F	68y	Alive	6y	Language problems: paraphasic errors	None known	Apathy	Language problems: logopenia, anomia, paraphasic errors, mutism	None known	*MRI*: moderate atrophy left frontal lobe, gliosis; *SPECT*: hypoperfusion left temporoparietal lobe	PPA
EII3	Not performed	M	Not known	73y	NA	Cognitive impairment and behavioral problems	Referred memory impairment	Aggressivness	None known	NA	NA	Clinical history was referred by relatives. Probable dementing syndrome
EII5	Negative	M	75y	84y, cardiac arrest	9y	Behavioral problems, aggressivness	None known	Aggressiveness	Paraphasic errors, aphasia, mutism	NA	*MRI*: diffuse cortical atrophy, ( +) anterior frontotemporal lobes	bvFTD

*AAO* age at onset, *AAD* age at death, *AD* Alzheimer’s disease, *PPA* primary progressive aphasia, *bvFTD* behavioral frontotemporal dementia, *MRI* magnetic resonance imaging, *SPECT* single photon emission computed tomography, *DTR* deep tendon reflex, *EMG* electromyography, *Y* years, *NA* not available, *F* female, *M* male.

### Family E

Affected members of Family E display a heterogeneous clinical picture, ranging from probable AD, to bvFTD and PPA. The clinical features of the affected members are summarized in Table 4.

The proband of the family deceased at 65 years of age, due to pneumonia and could not be included in the genetic screening. However, relatives described the patient presenting with a dementing syndrome with cognitive and memory impairment.

#### EII1

At 71 years of age, the patient presented with deficits in short-term memory and attention. No behavioral changes or language impairment were reported. Mild personality fluctuations appeared only during the latter course of the disease. At 76 years of age, the patient was diagnosed with probable AD and later with FTD with memory onset. She was disoriented and died of pneumonia at 80 years of age.

#### EII2

Language impairment, progressively worsening with paraphasic errors, characterizes the onset of symptoms in patient EII2, aged 68 years. Two years later, at the age of 70 years, the patient was diagnosed with PPA; the disease gradually evolved to include apathy and mutism. A MRI scan revealed gliosis and modest atrophy accentuated in the left frontal lobe. A functional imaging using Technetium Tc 99 m single-photon emission computed tomography (SPECT) showed hypoperfusion particularly in the temporoparietal lobe, on the left hemisphere.

#### EII5

At the age of 75 years, the patient developed a change in personality with aggressive behavior. After three years, he displayed language problems worsening to include mutism and aphasia. After nine years of disease, the patient died due to a cardiac arrest. An MRI scan revealed diffuse cortical atrophy particularly marked in the anterior frontotemporal lobes.

## Discussion

We carried out exome sequencing in 100 familial and apparently sporadic patients with FTD-like spectrum disorders and screened dementia and NCL genes.

Among the dementia genes, we identified 3 likely pathogenic variants in *GRN* in 3 sporadic cases (p.C105Y, p.C389fs, p.C139R), one *C9orf72* expansion in one sporadic case, 2 *CSF1R* mutations in the TK flanking regions and one loss of function mutation in *SORL1* (p.R744X) in 2/3 affected members of Family H. Additionally, we detected a novel putative LoF mutation in a NCL gene, *CLCN6* p.S116P, segregating with FTD with memory onset and PPA in Family E (Table 2).

We recently reported a *GRN* novel splice site mutation, *GRN* c.709-2A > T, in a multigenerational family from the same geographic area^37^ in Apulia and in this cohort identified only 3 moderately to frankly pathogenic mutations in 3 apparently sporadic bvFTD cases and showed that *GRN* mutations may account for only a minority of FTD cases (6.4%), in contrast to the high prevalence of *GRN* mutations that have been described in a cohort of the nearby Calabria region, where the overall contribution of *GRN* mutations was 53% (17/32) increasing to 71.4% in patients with family history of dementia (15/21)^38^. Analogously, the frequency of *C9orf72* expansions (1%) is much lower than the ones reported in other European countries and Italy particularly (6%)^39^ and this is likely not related to the North–South axis as the detected prevalence in Germany was 4.82% and, on the other hand, in Spain 25.49%^39^. This may further point to the isolation of these villages.

Interestingly, we reported 2 mutations in *CSF1R* in the TK domain flanking regions (aa 538–581 and 911–972): p.E573K and p.R549H, detected in 2 apparently sporadic patients with late-onset bvFTD. Although mutations in the TK regions of *CSF1R* (exons 12–22, aa 582–910) are causative for hereditary diffuse leukoencephalopathy with spheroids (HDLS), which clinically manifests as early-onset bvFTD-like with additional Parkinsonism, extrapyramidal or pyramidal signs^40^, also mutation in the *CSF1R* TK flanking regions have been already associated to early onset PPA^28^ and particularly p.E573K leads to a partial loss of the kinase activity and has been reported in a patient with ischemic embolic stroke without the typical clinical features of HDLS^29^, suggesting that missense mutations in the TK flanking regions leading to only a decreased TK activity may cause a milder phenotype compared to HDLS.

Among the dementia genes we detected a loss of function mutation in the VPS10 of *SORL1* (Aa 124–757), p.R744X, in 2/3 affected members of Family H displaying late-onset AD with severe language impairment and PPA with pyramidal signs. This mutation was also found in an asymptomatic at risk member of the third generation (HIII2), aged 42 years (average age at onset in Family H is 68 years) and was not detected in another familial member, displaying FTD with memory onset, suggesting that *SORL1* (p.R744X) may influence AD with language problems and PPA and that there may be additional genetic modifiers responsible for different phenotypic manifestations.

Importantly*, SORL1* variants clustering in the VPS10 domain have been reported as pathogenic and to segregate within AD families^41^ particularly with extrapyramidal signs like parkinsonism^42^ and language impairment^43^ and also to vascular dementia^44^ and small vessel disease^45^. Therefore, our finding may support the role of *SORL1* influencing motor function and language skills in dementing disorders.

Finally we report a near splice site mutation in *CLCN6*, p.S116P, segregating with an heterogeneous phenotype (bvFTD, FTD with memory onset and PPA) in Family E.

This mutation has been also reported in an asymptomatic member in the third generation (EIII3) that , aged 52 years, may manifest the phenotype later in life (average age at onset for the affected members was 71.3 years).

*CLCN6* encodes for the protein CIC-6, a Cl^-^ channel protein that is almost exclusively expressed in neurons. It co-localizes with late endosomes and mediates the exchange of endosomal Cl^-^ for cytosolic H^+^^46^. It is plausible that this putative loss of function mutation may lead to a less efficient late endosomal acidification, thus compromising the protein degradation and the autophagosomal pathway, which are pH dependent. Therefore, it may affect TDP-43 degradation, contributing to its cytoplasmatic deposition.

Importantly, in vivo studies with *Clcn6*^-/-^ mice recapitulate some of the histological and clinical features of late-onset NCL, characterized by the accumulation of storage material (saposinB, lamp-1, cathepsin D and lysosomal acid phosphatase) in the lysosomal system, leading to mild cognitive impairment and behavioral abnormalities^46^. Remarkably, a growing number of studies has shown that NCL and FTD may share common pathogenic mechanisms. First, *GRN* heterozygous LoF mutations cause FTD whereas homozygous LoF mutations cause NCL^10,47^.

Second, heterozygous mutations in the Cathepsin F (*CTSF*) gene, that in homozygosity are causative for adult-onset NCL, have been recently reported in a patient with early-onset FTD and motor symptoms^9^.

Third, NCL is characterized by pathological alterations typical of FTD and vice versa: NCL presents a different degree of TDP-43 phosphorylation and GRN-associated FTD is characterized by the elevation of lysosomal proteins and accumulation of saposin B, subunit c of mitochondrial ATP synthase (SCMAS), ubiquitin and p62 protein^48^. Fourth, *TMEM106B*, *VCP*, *CHMP2B* and *SORT1*, harbor variants identified as disease causing or risk factors for FTD and seem to play a role in endosomal trafficking^49–52^. As with *CLCN6*, *TMEM106B* and *CHMP2B* co-localize to the late endosomes and appear to be involved in the endosome-lysosome fusion. This represents a critical step for the autophagosome-mediated degradation of proteins and may be involved in TDP-43 turnover. Moreover, *CLCN6* has been associated to increased levels of N-terminal cleavage product of the B-type natriuretic peptide (NT-proBNP), a well-established biomarker for dementia^53,54^.

The strength of our study relies on the enormous advantage of performing a genetic analysis in a very inbred FTD cohort from geographically and historically isolated areas and therefore enriched for rare alleles with high penetrance and strong effect size. On the other hand, a limitation of our study is represented by the lack of multigenerational and expanded families to analyze the segregation of rare pathogenic alleles.

Our study includes *SORL1* VPS mutations, *CSF1R* and *CLCN6* in the genetic spectrum associated to dementing syndromes with frontal signs, memory deficits, language impairment and pyramidal signs and in concert with a growing body of evidence supports the potential shared pathogenic ground underpinning FTD-like disorders and adult-onset neuronal ceroid-lipofuscinosis.

## Supplementary information

Supplementary information.

## Data Availability

All data generated or analysed during this study are included in this published article (and its Supplementary Information files).
