# Supplementary material for: Exploring dementia and neuronal ceroid lipofuscinosis genes in 100 FTD-like patients from 6 towns and rural villages on the Adriatic Sea cost of Apulia
Source: Sci Rep. 2021 Mar 18;11:6353. doi: 10.1038/s41598-021-85494-x (PMC7973810; doi:10.1038/s41598-021-85494-x)
Supplement: Supplementary file 1 — Supplementary information. [file 41598_2021_85494_MOESM1_ESM.docx]

Exploring dementia and neuronal ceroid lipofuscinosis genes in 100 FTD-like patients from 6 towns and rural villages on the Adriatic sea cost of Apulia

Celeste Sassi^a^, Rosa Capozzo^b,^  Monia Hammer^a^, Chiara Zecca^b^, Monica Federoff^a^, Cornelis Blauwendraat ^a^ Nick Bernstein^a^, Jinhui Ding^a^, J. Raphael Gibbs^a^, Timothy Price^a^, Andrew Singleton^a^ and Giancarlo Logroscino^b,c^

^a^ Laboratory of Neurogenetics, National Institute on Aging, National Institutes of Health, Bethesda, MD, USA

^b^ Department of Clinical Neurology and Research, University of Bari, “Pia Fondazione Cardinale G. Panico”, Tricase (LE), Italy

^c^ Nurodegenerative Diseases Unit, Department of Basic Medical Science, Neuroscience and Sense Organs, University of Bari, Italy

**Correspondence**:

Celeste Sassi, MD, PhD
Charité – Universitätsmedizin Berlin

Charitéplatz 1

D-10117 Berlin

Tel.: 030 – 450 560 149

Fax: 030 – 450 560 915

[celeste.sassi@charite.de](mailto:celeste.sassi@charite.de)

| **Gene** | **Mutation** | **Location** | **Type of inheritance** | **Phenotype** | **Ref.** |
| --- | --- | --- | --- | --- | --- |
| *GRN* | g.10325_10331delCTGCTGT, Cys157LysfsX97 | South Italy, Neaples | 3 familial cases, segregation with FTD | clinical heterogeneity: bvFTD und nfvPPA | ^1^ |
| *GRN* | g.101349_101355delCTGCTGT, C157KfsX97 | Southern Italy, Neaples | 7 Italian patients, 60% Fam | 5 bvFTD and 2 CBS | ^2^ |
| *GRN* | p. Leu271LeufsX10 | Northern Italy: Brescia | 2 Families | FTDP-17 to corticobasal syndrome | ^3^ |
| *GRN* | p.Tyr272SerfsX10 | Northern Italy: Milan und Brescia | 4 families | bvFTD and PNFA | ^4^ |
| *GRN* | p. Thr272fs | Northern Italy: Milan, Brescia and Turin | 9/14 families, autosomal dominant | 80% bvFTD und 20% PPA | ^5^ |
| *GRN* | c.709-2 A>T | Southern Italy, Tricase | 1 large Family, AD | bvFTD, PNFA, FTD with memory problem | ^6^ |
| *GRN* | c.1145insA, p.A266P, p.C126W | Southern Italy: Lamezia Terme | Familial and Sporadic cases | bvFTD |  |
| *UBQLN2* | p.M446R, P497H, P506S, P533L | Italy | Familial and Sporadic cases | EO ALS-FTD | ^7^ |
| *C9ORF72* |  | Italy | 23.9% FALS and 5.2% of SALS, C9orf72 expansion was associated with higher OR of bulbar onset, FTD diagnosis | ALS-FTD | ^8^ |
| *MAPT* | TAU gene mutations | Northern Italy: Brescia | 7.6% familial FTD (38 patients) | familial FTD | ^9^ |
| *VCP* | p.R155C | Rome | 1 Family | autosomal-dominant hereditary inclusion-body myopathy associated | ^10^ |
| *VCP* | p.R159C | Northern Italy: Milan | 1 sporadci case | IBMPFD: FTD, IBM and Paget’s disease | ^11^ |
| *VCP* | R155H | Northern Italy: Genoa | 1 Family | IBMPFD: FTD, IBM and Paget’s disease | ^12^ |
| *PRNP* | P102L | Northern Italy: Milan |  | atypical frontotemporal dementia,  whereas two other members showed typical cerebellospinal  symptoms. | ^13^ |
| *PRNP* | p.P39L | Italy | 1/761 patients with FTD (0.13%) | FTD with apathetic phenotype | ^14^ |
| *TREM2* | p.Thr66Met in homozygosity | Italy | consanguineous Italian family (2 sibs) | FTLD with parkinsonism and epilepsy | ^15^ |

**Table S1**. Mutations in FTD Mendelian genes and *C9orf72* expansions reported in Italian sporadic or familial cases

| **Gene** | **Transcript** | **Phenotype** |
| --- | --- | --- |
| *GRN* | NM_002087 | bvFTD, PPA, NCL 11 |
| *MAPT* | NM_001123066 | FTD, PSP, DLB |
| *VCP* | NM_007126 | FTD |
| *C9ORF72* | NM_001256054 | FTD and FTD-ALS |
| *TREM2* | NM_001271821 | FTD, AD |
| *TYROBP* | [NM_003332](http://www.ncbi.nlm.nih.gov/nuccore/NM_003332.4) | FTD |
| *UBQLN2* | NM_013444 | FTD |
| *PRNP* | NM_000311 | PRION DISEASES |
| *APP* | NM_000484 | AD, CAA |
| *PSEN1* | NM_000021 | AD, CAA |
| *PSEN2* | NM_000447 | AD |
| *SORL1* | NM_003105 | AD |
| *CSF1R* | NM_001288705 | HDLS |
| *NOTCH3* | NM_000435 | CADASIL |
| *SNCA* | NM_001146055 | DLB, PD |
| *GBA* | NM_001171811 | DLB, PD, Gaucher Disease |
| *CLN10/CTSD* | NM_001909 | NCL (Ceroid Lipofuscinosis neuronal 10) |
| *CTSF* | [NM_003793](http://www.ncbi.nlm.nih.gov/nuccore/NM_003793) | NCL (Ceroid Lipofuscinosis neuronal 13) |
| *CLN1/PPT1* | NM_001142604 | NCL (Ceroid Lipofuscinosis neuronal 1) |
| *CLN2/TPP1* | NM_000391 | NCL (Ceroid Lipofuscinosis neuronal 2), SCA7 |
| *CLN3* | NM_001286105 | NCL (Ceroid Lipofuscinosis neuronal 3) |
| *CLN5* | NM_006493 | NCL (Ceroid Lipofuscinosis neuronal 5) |
| *CLN6* | NM_017882 | NCL (Ceroid Lipofuscinosis neuronal 6) |
| *CLN7/MFSD8* | NM_152778 | NCL (Ceroid Lipofuscinosis neuronal 7) |
| *CLN4* | [NM_025219](http://www.ncbi.nlm.nih.gov/nuccore/NM_025219) | NCL (Kufs disease) |
| *CLCN6* | NM_001286 | NCL mild forms of human neuronal ceroid lipofuscinosis |
| *CLCN7* | [NM_001287](http://www.ncbi.nlm.nih.gov/nuccore/NM_001287) | NCL mild forms of human neuronal ceroid lipofuscinosis |
| *SGSH* | NM_000199 | NCL Mucopolysaccharidosis type IIIA |

**Table S2.** List of Mendelian dementia and neurolipofuscinosis genes selected in the current study. BvFTD, behavioural FTD; PPA, primary progressive aphasia; NCL, neuroceroidlipofuscinosis; PSP, progressive supranuclear palsy; DLB, dementia with Lewy bodies; AD, Alzheimer’s disease; CAA, cerebral amyloid angiopathy; HDLS, hereditary diffuse leukodystrophy with spheroids; CADASIL, cerebral autosomal dominant arteriopathy with subcortical infarcts and leukoencephalopathy; PD, Parkinson disease.

| GENE | POSITION | NUCLEOTIDE  CHANGE | Aa  CHANGE | TRANSCRIPT | ExAC | MUTATION TASTER | POLYPHEN2 | SIFT | EST BRAIN | Grantham  S. | PhastCons  S. | GERP  S. | References |
| --- | --- | --- | --- | --- | --- | --- | --- | --- | --- | --- | --- | --- | --- |
| *INSRR,NTRK1* | chr1:156821804 | c.817 G>A | p.R273C | NM_014215 |  | disease_causing | Probably damaging (1) | damaging (0.05) | NOT EXPRESSED IN THE BRAIN | 180 | 1 | 4.62 |  |
| *ACTN2* | chr1:236924423 | c.2476 G>A | p.D826N | NM_001103.3 |  | disease_causing | Probably damaging (1) | damaging (0) | 39 TPM  (944 MAX) | 23 | 0.892 | 6.03 |  |
| *FLG,FLG-AS1* | chr1:152286278 | c.1084 C>T | p.E362K | NM_002016.1 |  | polymorphism | Probably damaging (0.998) | tolerated (0.21) | 107 TPM  (488 MAX) | 56 | 0.001 | 1.23 |  |
| *H6PD* | chr1:9324293 | c.1741 C>T | p.R581C | NM_004285.3 | 0.00001881 | disease_causing | Probably damaging (0.996) | damaging (0.01) | 14 TPM  (155 MAX) | 180 | 0.979 | 5.67 |  |
| *PRAMEF1* | chr1:12854513 | c.737 C>T | p.T246M | NM_023013.2 |  | polymorphism | possibly damaging (0.933) | tolerated (0.23) | NOT EXPRESSED IN THE BRAIN | 81 | 0 | 0.34 |  |
| *TNR* | chr1:175360441 | c.1490 G>A | p.S497L | NM_003285.2 |  | disease_causing | possibly damaging (0.816) | tolerated (0.23) | 20 TPM  (20 MAX) | 145 | 0.989 | 5.23 | ^1, 2, 3, 4, 5, 6^ |
| *HSPG2* | chr1:22198765 | c.4135 C>T | p.G1379S | NM_005529.5 |  | disease_causing | possibly damaging (0.546) | tolerated (0.26) | 29 TPM  (836 MAX) | 56 | 0.655 | 5.15 |  |
| *PADI3* | chr1:17593247 | c.442 G>A | p.G148S | NM_016233.2 | 0.00003295 | disease_causing | benign (0.357) | damaging (0) | 10 TPM  (24 MAX) | 56 | 0.655 | 5.15 |  |
| *ZNF281* | chr1:200378565 | c.269 G>T | p.P90H | NM_001281293.1 | 0.001096 | polymorphism | benign (0.172) | damaging (0) | 51 TPM  (248 MAX) | 77 | 0.998 | 3.54 |  |
| *AHDC1* | chr1:27876321 | c.2306 G>A | p.A769V | NM_001029882.2 | | polymorphism | benign (0.16) | tolerated (0.32) | 15 TPM  (160 MAX) | 64 | 0.049 | 1.77 |  |
| *ZNF683* | chr1:26691667 | c.370 T>G | p.T124P | NM_001114759.1 | 0.00002417 | polymorphism | benign (0.069) | tolerated (0.13) | 8 TPM (89 MAX) | 38 | 0.713 | 2.63 |  |
| *SH3D21* | chr1:36785786 | c.1522 T>A | p.S508T | NM_001162530.1 | | polymorphism | benign (0.031) | tolerated (0.19) | NOT EXPRESSED IN THE BRAIN | 58 | 0 | 0.722 |  |
| *TAS1R2* | chr1:19166245 | c.2368 T>C | p.I790V | NM_152232.2 | 0.00004127 | polymorphism | benign (0.012) | tolerated (0.34) | NA | 29 | 0.98 | 2.63 |  |
| *MAP10* | chr1:232942208 | c.1439 C>T | p.P480L | NM_019090.2 | 0.000008312 | polymorphism | benign (0.012) | tolerated (0.62) | 7 TPM (60 MAX) | 98 | 0 | -0.218 |  |
| *CREB3L4* | chr1:153941827 | c.439 T>C | p.F147L | NM_001255978.1 | | polymorphism | benign (0.002) | tolerated (0.69) | 12 TPM (174 MAX) | 22 | 0.002 | 0.416 |  |
| *ZNF142* | chr2:219508654 | c.2585 C>A | p.R862L | NM_001105537.2 | 0.000008285 | polymorphism | Pbably damaging (0.997) | tolerated (0.23) | 29 TPM  (217 MAX) | 102 | 1 | 4.2 |  |
| *C2orf49* | chr2:105959375 | c.337 A>G | p.I113V | NM_024093.1 | 0.00001648 | disease_causing | Pbably damaging (0.99) | tolerated (0.21) | 21 TPM  (89 MAX) | 29 | 1 | 5.73 |  |
| *TRAPPC12* | chr2:3428333 | c.1316 G>A | p.G439D | NM_016030.5 |  | disease_causing | benign (0.04) | tolerated (0.51) | 48 TPM  (595 MAX) | 94 | 1 | 5.6 |  |
| *RNF25* | chr2:219530758 | c.454 G>A | p.P152S | NM_022453.2 | 0.000008245 | polymorphism | benign (0.011) | tolerated (0.25) | 21 TPM  (121 MAX) | 74 | 0.374 | 3.14 |  |
| *EVA1A* | chr2:75745233 | c.34 C>T | p.V12M | NM_001135032.1 | 0.000165 | polymorphism | benign (0.001) | damaging (0.02) | NOT EXPRESSED IN THE BRAIN | 21 | 0 | -10.2 |  |
| *LNP1* | chr3:100148685 | c.112 C>T | p.R38X | NM_001085451.1 | 0.000008291 | disease_causing | NA | na | 34 TPM  (90 MAX) | NA | 0 | 0.177 |  |
| *SLCO2A1* | chr3:133673824 | c.611 G>A | p.S204L | NM_005630.2 | 0.00004145 | disease_causing | Probably damaging (1) | damaging (0) | 9 TPM  (173 MAX) | 145 | 0.972 | 5.89 |  |
| *ACOX2* | chr3:58517449 | c.674 C>T | p.R225Q | NM_003500.3 | 0.0001157 | disease_causing | Pbably damaging (0.999) | damaging (0) | 4 TPM  (145 MAX) | 43 | 1 | 4.66 |  |
| *CNTN3* | chr3:74420498 | c.507 A>C | p.D169E | NM_020872.1 | 0.000008271 | disease_causing | possibly damaging (0.839) | damaging (0) | 13 TPM  (62 MAX) | 45 | 0.991 | 0.79 |  |
| *AFP* | chr4:74308135 | c.605 T>C | p.F202S | NM_001134.1 | 0.0000165 | disease_causing | Pbably damaging (0.994) | damaging (0) | NOT EXPRESSED IN THE BRAIN | 155 | 1 | 5.33 |  |
| *ATP8A1* | chr4:42627694 | c.201 T>G | p.R67S | NM_001105529.1 | | disease_causing | Pbably damaging (0.967) | tolerated (0.07) | 89 TPM  (339 MAX) | 110 | 0.993 | 0.637 |  |
| *BVES* | chr6:105572523 | c.547 C>A | p.V183F | NM_001199563.1 | 0.000008274 | disease_causing | Probably damaging (1) | damaging (0.03) | 9 TPM  (242 MAX) | 50 | 1 | 5.86 |  |
| *SYNJ2* | chr6:158454651 | c.650 G>A | p.R217H | NM_003898.3 | 0.00002479 | disease_causing | Probably damaging (1) | damaging (0.01) | 69 TPM  (121 MAX) | 29 | 1 | 3.57 |  |
| *BEND3* | chr6:107391955 | c.440 C>T | p.G147E | NM_001080450.2 | 0.000008242 | polymorphism | benign (0.244) | damaging (0.01) | NOT EXPRESSED IN THE BRAIN | 98 | 0.486 | 3.99 |  |
| *PHTF2* | chr7:77549628 | c.707 C>T | p.S236L | NM_001127357.1 | | disease_causing | Probably damaging (1) | tolerated (0.64) | 21 TPM  (197 MAX) | 145 | 1 | 6.06 |  |
| *AP5Z1* | chr7:4830898 | c.2306 C>G | p.T769R | NM_014855.2 | 0.00004335 | disease_causing | Pbably damaging (0.998) | damaging (0) | 15 TPM  (158 MAX) | 71 | 0.517 | 5.13 | ^7, 8^ |
| *ZNF786* | chr7:148768478 | c.1386 G>T | p.F462L | NM_152411.3 | 0.00003403 | diseae_causing | Pbably damaging (0.984) | damaging (0.01) | 4 TPM (28 MAX) | 22 | 0.015 | 2.29 |  |
| *SORL1* | chr11:121421343 | c.2230 C>T | p.R744X | NM_003105.5 |  | disease_causing | NA | na | 122 TPM  (367 MAX) | NA | 0.181 | 4.45 |  |
| *CADPS2,RNF148* | chr7:122342290 | c.515 T>C | p.Y172C | NM_198085.1 | 0.000008281 | polymorphism | possibly damaging (0.808) | tolerated (0.11) | 44 TPM  (107 MAX) | 194 | 0.999 | 2.54 |  |
| *VWDE* | chr7:12409408 | c.2524 C>T | p.V842I | NM_001135924.1 | | polymorphism | benign (0.296) | tolerated (0.32) | NOT EXPRESSED IN THE BRAIN | 29 | 0.997 | 2.07 |  |
| *PXDNL* | chr8:52321834 | c.2350 C>G | p.A784P | NM_144651.4 | 0.0007003 | polymorphism | possibly damaging (0.896) | damaging (0) | NOT EXPRESSED IN THE BRAIN | 27 | 0.004 | 2.53 |  |
| *PABPC1* | chr8:101721705 | c.1227 G>T | p.F409L | NM_002568.3 |  | disease_causing | benign (0.094) | tolerated (0.09) | 163 TPM  (3110 MAX) | 22 | 1 | 4.5 |  |
| *PTPN3* | chr9:112172595 | c.1279 C>T | p.G427S | NM_001145369.1 | 0.00003295 | disease_causing | Pbably damaging (0.997) | tolerated (0.78) | 73 TPM  (242 MAX) | 56 | 0.348 | 5.67 |  |
| *CTSV* | chr9:99799777 | c.247 T>C | p.M83V | NM_001201575.1 | 0.00004947 | disease_causing | Pbably damaging (0.961) | damaging (0) | NA | 21 | 1 | 3.81 |  |
| *FCN1* | chr9:137801657 | c.968 A>G | p.V323A | NM_002003.3 | 0.0008002 | polymorphism | benign (0.122) | damaging (0.03) | NOT EXPRESSED IN THE BRAIN | 64 | 0.987 | 3.2 |  |
| *MRPL50* | chr9:104160817 | c.58 C>G | p.G20R | NM_019051.2 |  | polymorphism | benign (0.011) | tolerated (0.88) | 35 TPM (  803 MAX) | 125 | 0 | -2.44 |  |
| *OR1Q1* | chr9:125377047 | c.31 C>T | p.H11Y | NM_012364.1 |  | polymorphism | benign (0.001) | damaging (0) | NA | 83 | 0.466 | -1.14 |  |
| *ENTPD1,ENTPD1-AS1* | chr10:97607399 | c.1031 A>G | p.Y344C | NM_001098175.1 | | polymorphism | Pbably damaging (0.996) | tolerated (0.08) | 29 TPM  (242 MAX) | 194 | 0.018 | 0.086 |  |
| *USP54* | chr10:75258490 | c.4952 G>C | p.S1651C | NM_152586.3 | 0.000008335 | polymorphism | possibly damaging (0.934) | damaging (0.05) | 39 TPM  (255 MAX) | 112 | 0.001 | 0.988 |  |
| *JMJD1C* | chr10:64974815 | c.455 T>C | p.N152S | NM_004241.2 |  | disease_causing | possibly damaging (0.857) | tolerated (0.31) | 56 TPM  (213 MAX) | 46 | 1 | 4.23 |  |
| *PCDH15* | chr10:55582870 | c.4637 T>A | p.N1541I | NM_001142764.1 | | polymorphism | benign (0.295) | damaging (0) | 10 TPM  (64 MAX) | 149 | 0.003 | -1.28 |  |
| *ANKRD2* | chr10:99332496 | c.32 G>A | p.G11D | NM_001129981.1 | | polymorphism | benign (0.002) | damaging (0) | 2 TPM  (864 MAX) | 94 | 0.207 | 2.92 |  |
| *SERGEF* | chr11:18010279 | c.709 C>A | p.G237X | NM_012139.2 | 0.00000826 | disease_causing | NA | na | 49 TPM  (235 max) | NA | 1 | 5.72 |  |
| *CD12* | chr11:104763149 | c.341 C>A | p.R114L | NM_001191016.1 | | polymorphism | Probably damaging (1) | damaging (0) | NOT EXPRESSED IN THE BRAIN | 102 | 0.002 | 3.57 |  |
| *MMP10* | chr11:102642772 | c.1301 G>C | p.P434R | NM_002425.2 | 0.00000825 | polymorphism | possibly damaging (0.799) | tolerated (0.46) | NOT EXPRESSED IN THE BRAIN | 103 | 0.001 | 0.591 |  |
| *JRKL* | chr11:96125212 | c.1399 G>A | p.E467K | NM_001261833.1 | 0.00001664 | polymorphism | benign (0) | tolerated (0.25) | NOT EXPRESSED IN THE BRAIN | 56 | 0.472 | 2.87 |  |
| *SOX5* | chr12:23757410 | c.1036 C>G | p.G346R | NM_001261414.1 | 0.00003298 | disease_causing | possibly damaging (0.948) | damaging (0.02) | 32 TPM (52MAX) | 125 | 1 | 6.16 | ^9,10^ |
| *RBMS2* | chr12:56982109 | c.1094 C>T | p.A365V | NM_002898.3 | 0.000008237 | disease_causing | possibly damaging (0.818) | tolerated (0.11) | 25 TPM  (407 MAX) | 64 | 0.902 | 3.88 |  |
| *OAS3* | chr12:113398878 | c.1660 C>A | p.Q554K | NM_006187.2 | 0.0000286 | polymorphism | benign (0.092) | tolerated (0.24) | NOT EXPRESSED IN THE BRAIN | 53 | 0.426 | 2.56 |  |
| *CCNT1* | chr12:49088075 | c.922 T>C | p.S308G | NM_001240.3 | 0.00001648 | polymorphism | benign (0) | tolerated (0.29) | 12 TPM  (75 MAX) | 56 | 0.998 | 3.78 |  |
| *OXA1L* | chr14:23239450 | c.812 T>G | p.I271S | NM_005015.3 |  | disease_causing | possibly damaging (0.952) | damaging (0) | 29 TPM (598) | 142 | 0.24 | 5.99 |  |
| *PATL2* | chr15:44962061 | c.790 C>T | p.E264K | NM_001145112.1 | 0.0001012 | disease_causing | benign (0.443) | damaging (0.03) | NOT EXPRESSED IN THE BRAIN | 56 | 0.993 | 5.74 |  |
| *MAPK8IP3* | chr16:1816767 | c.2962 C>T | p.H988Y | NM_001040439.1 | 0.000008514 | disease_causing | Pbably damaging (0.991) | damaging (0.04) | 44 TPM  (193 MAX) | 83 | 1 | 4.03 |  |
| *CLUAP1* | chr16:3569946 | c.623 C>T | p.S208F | NM_015041.2 | 0.000008258 | disease_causing | Pbably damaging (0.988) | na | 39 TPM  (89 MAX) | 155 | 1 | 4.38 |  |
| *ZZEF1* | chr17:3992103 | c.2110 G>A | p.R704W | NM_015113.3 |  | disease_causing | Probably damaging (1) | damaging (0.01) | 42 TPM  (435 MAX) | 101 | 1 | 4.93 |  |
| *STAC2* | chr17:37373367 | c.457 A>G | p.S153P | NM_198993.3 | 0.00001652 | disease_causing | Pbably damaging (0.994) | damaging (0.04) | 17 TPM  (95 MAX) | 74 | 1 | 5.18 |  |
| *KRT12* | chr17:39019429 | c.1262 C>T | p.R421H | NM_000223.3 |  | disease_causing | possibly damaging (0.589) | tolerated (0.18) | 10 TPM  (1589 MAX) | 29 | 1 | 4.07 |  |
| *ZBTB4* | chr17:7366325 | c.1976 C>T | p.G659E | NM_001128833.1 | | polymorphism | benign (0.181) | tolerated (0.77) | 73 TPM  (339 MAX) | 98 | 0.999 | 4.1 |  |
| *LGALS9* | chr17:25970641 | c.535 C>A | p.Q179K | NM_009587.2 |  | polymorphism | benign (0.116) | tolerated (0.67) | 14 TPM  (206 MAX) | 53 | 0.118 | 1.41 |  |
| *ST6GALNAC2* | chr17:74569305 | c.502 T>C | p.N168D | NM_006456.2 |  | polymorphism | benign (0.004) | tolerated (0.44) | 10 TPM  (99 MAX) | 23 | 0.004 | -3.91 |  |
| *ZNF845* | chr19:53856702 | c.2774 G>A | p.R925H | NM_138374.1 | 0.008272 | polymorphism | Pbably damaging (0.973) | tolerated (0.23) | 3 TPM  (117 MAX) | 29 | 0 | -4 |  |
| *ETHE1* | chr19:44030450 | c.278 G>A | p.S93F | NM_014297.3 | 0.0003393 | polymorphism | possibly damaging (0.855) | damaging (0.02) | 16 TPM  (145 MAX) | 155 | 0.036 | 4.86 |  |
| *MYH7B* | chr20:33575386 | c.1300 G>C | p.G434R | NM_020884.3 |  | disease_causing | Probably damaging (1) | damaging (0) | 4 TPM  (275 MAX) | 125 | 1 | 3.53 |  |
| *PREX1* | chr20:47249171 | c.4274 T>C | p.N1425S | NM_020820.3 |  | polymorphism | benign (0.004) | tolerated (0.29) | 58 TPM  (376 MAX) | 46 | 0.225 | 2.35 |  |
| *IL17RA* | chr22:17589953 | c.1844 C>T | p.T615I | NM_014339.6 | 0.00001849 | polymorphism | benign (0.497) | tolerated (0.12) | 18 TPM  (139 MAX) | 89 | 0 | 1.77 |  |
| *DGCR2* | chr22:19044633 | c.545 G>A | p.S182L | NM_001173533.1 | 0.0006876 | polymorphism | benign (0.355) | tolerated (0.72) | 695 TPM  (695 MAX) | 145 | 0.845 | 5.56 |  |
| *TXLNG* | chrX:16859560 | c.862 C>T | p.R288C | NM_001168683.1 | 0.0000612 | disease_causing | Probably damaging (1) | tolerated (0.17) | 20 TPM  (155 MAX) | 180 | 0.998 | 5.07 |  |
| *ALG13* | chrX:110924456 | c.10 G>A | p.V4M | NM_001099922.2 | | polymorphism | possibly damaging (0.91) | damaging (0.03) | 36 TPM  (970 MAX) | 21 | 0.191 | -0.007 |  |

**Table S3**. List of novel nonsynonymous coding variants segregating within **Family H**. In light blue are the variants which meet all the filter criteria: 1) novel variants; 2) segregating with the disease; 3) predicted as damaging by at least 2 out of 3 in silico prediction softwares (MUTATION TASTER, POLYPHEN2, SIFT) and 4) highly expressed in the brain and highly conserved (Grantham > 50, PhastCons > 0.4 and GERP >4). The other variants do not meet at least one of the filter criteria. In orange, variants in genes which may contribute to the disease phenotype. Chr, chromosome ; NA, not available.

| **GENE** | **POSITION** | **NUCLEOTIDE** | **Aa**  **CHANGE** | **rs number** | **ExAC** | **MUTATION TASTER** | | **POLYPHEN2** | **SIFT** | **EST BRAIN** | **Grantham** | **PhastCons** | **GERP** |
| --- | --- | --- | --- | --- | --- | --- | --- | --- | --- | --- | --- | --- | --- |
|  |  | **CHANGE** |  |  |  |  |  |  |  |  |  |  |  |
| *UBR4* | chr1:19404529 | c.15265 C>T | p.A5089T | NM_020765.2 |  | polymorphism | | Benign (0.001) | tolerated (0.34) | 121 TPM (767 MAX) | 58 | 0.452 | 3.99 |
| *CCDC30* | chr1:43042699 | c.864 T>G | p.I288M | NM_001080850.2 | 0.000008666 | polymorphism | | Probably damaging (0.97) | tolerated (0.15) | 5 TPM (60 MAX) | 10 | 1 | 3.48 |
| *CLCN6* | chr1:11879611 | c.280 T>C | p.S94P | NM_001256959.1 |  | disease_causing | | Possibly damaging (0.958) | tolerated (0.2) | 58 TPM (193 MAX) | 74 | 0.913 | 5.34 |
| *CROCC* | chr1:17250973 | c.350 G>A | p.R117K | NM_014675.3 | 0.00004369 | disease_causing | | Probably damaging (0.996) | tolerated (0.15) | 19 TPM (64 MAX) | 26 | 1 | 4.99 |
| *KANSL1L* | chr2:210940376 | c.1655 G>C | p.S552C | NM_152519.2 | 0.0001651 | disease_causing | | Probably damaging (1) | damaging (0.02) | 21 TPM (128 MAX) | 112 | 1 | 5.56 |
| *BARD1* | chr2:215593563 | c.2171 G>A | p.A724V | NM_000465.2 | 0.00004121 | disease_causing | | Probably damaging (1) | damaging (0.01) | NOT EXPRESSED IN THE BRAIN | 64 | 0.871 | 5.81 |
| *WM8* | chr2:234904975 | c.2945 C>T | p.T982M | NM_024080.4 |  | disease_causing | | Probably damaging (1) | damaging (0.01) | NOT EXPRESSED IN THE BRAIN | 81 | 0.978 | 5.61 |
| *GPC1* | chr2:241404059 | c.910 T>G | p.F304V | NM_002081.2 |  | disease_causing | | Benign (0.153) | damaging (0.01) | 53 TPM (188 MAX) | 50 | 1 | 3.98 |
| *OSBPL6* | chr2:179236854 | c.1364 C>T | p.A455V | NM_001201480.1 | 0.00001655 | disease_causing | | Probably damaging (0.985) | tolerated (0.18) | 26 TPM (77 MAX) | 64 | 1 | 5.55 |
| *COL8A1* | chr3:99509699 | c.173 T>C | p.V58A | NM_001850.4 | 0.0002966 | disease_causing | | Possibly damaging (0.924) | tolerated (1) | 2 TPM (466 MAX) | 64 | 1 | 5.73 |
| *PHC3* | chr3:169831283 | c.2218 C>G | p.E740Q | NM_024947.3 |  | disease_causing | | Possibly damaging (0.906) | damaging (0.02) | 21 TPM (248 MAX) | 29 | 1 | 5.51 |
| *VWA5B2* | chr3:183958672 | c.2834 C>G | p.T945S | NM_138345.1 |  | polymorphism | | Benign (0.018) | tolerated (1) | 16 TPM (91MAX) | 58 | 0.272 | 3.16 |
| *CLDN16* | chr3:190106027 | c.119 T>C | p.V40A | NM_006580.3 | 0.000008257 | polymorphism | | Benign (0.383) | damaging (0.01) | NOT EXPRESSED IN THE BRAIN | 64 | 0.229 | 2.31 |
| *CRIPAK* | chr4:1389029 | c.730 G>A | p.V244M | NM_175918.3 | 0.00001933 | polymorphism | | Probably damaging (0.998) | tolerated (0.13) | 27 TPM (99 MAX) | 21 | 0 | -0.188 |
| *SLBP* | chr4:1695364 | c.773 C>T | p.C258Y | NM_006527.2 | 0.00001648 | disease_causing | | Probably damaging (1) | damaging (0) | 32 TPM (109 MAX) | 194 | 0.72 | 4.47 |
| *PPRC1A* | chr4:23830051 | c.729 G>T | p.H243Q | NM_013261.3 |  | polymorphism | | Probably damaging (0.967) | tolerated (0.45) | 16 TPM (102 MAX) | 24 | 0.995 | 1.92 |
| *SDAD1* | chr4:76877181 | c.1963 G>A | p.R655W | NM_018115.2 | 0.00009068 | disease_causing | | Benign (0.24) | damaging (0.03) | 22 TPM (147 MAX) | 101 | 1 | 1.49 |
| *C4orf3* | chr4:120221566 | c.125 T>G | p.H42P | NM_001001701.3 |  | NA | | Benign (0.009) | tolerated (0.07) | 131 TPM (392 MAX) | 77 | 0.003 | -0.694 |
| *APBB3* | chr5:139941227 | c.713 C>T | p.C238Y | NM_006051.3 | 0.00001647 | polymorphism | | Benign (0) | damaging (0) | 150 TPM (321 MAX) | 194 | 0.998 | 2.48 |
| *AIM1* | chr6:106975266 | c.3275 T>C | p.I1092T | NM_001624.2 | 0.0004201 | polymorphism | | Benign (0.001) | tolerated (0.44) | 1 TPM (61 MAX) | 89 | 0.167 | 2.62 |
| *NMBR* | chr6:142396876 | c.1082 C>T | p.R361H | NM_002511.2 | 0.00004945 | | disease_causing | Benign (0.059) | TOLERATED (0.12) | 1 TPM (1 MAX) | 29 | 0.577 | 3.49 |
| *DBF4* | chr7:87537374 | c.1921 A>G | p.S641G | NM_006716.3 |  | | polymorphism | Benign (0.006) | DAMAGING (0.05) | 13 TPM (188 MAX) | 56 | 0.992 | 2.07 |
| *MCM7* | chr7:99695552 | c.362 T>A | p.Y121F | NM_001278595.1 |  | | disease_causing | Benign (0.001) | DAMAGING (0.01) | 442 TPM (1518 MAX) | 22 | 1 | 4.45 |
| *TMEM71* | chr8:133764233 | c.112 C>G | p.D38H | NM_001145153.1 | 0.00002495 | | polymorphism | Probably damaging (1) | DAMAGING (0) | 10 TPM (343 MAX) | 81 | 0.994 | 4.74 |
| *MYOM2* | chr8:2040209 | c.1864 C>T | p.R622X | NM_003970.2 |  | | disease_causing | NA | NA | 38 TPM (268 MAX) | NA | 0.117 | 2.45 |
| *SPATA31E1* | chr9:90502638 | c.3236 C>T | p.S1079F | NM_178828.4 | 0.000008257 | | polymorphism | Probably damaging (0.983) | DAMAGING (0.01) | NOT EXPRESSED IN THE BRAIN | 155 | 0 | -1.8 |
| *DDX31* | chr9:135505740 | c.1832 G>A | p.P611L | XM_005272206.1 | 0.00004943 | | disease_causing | Benign (0.03) | TOLERATED (0.6) | 5 TPM (82 MAX) | 98 | 0.79 | -4.3 |
| *HNRNPF* | chr10:43882752 | c.581 T>C | p.Y194C | NM_001098204.1 |  | | disease_causing | Probably damaging (0.97) | TOLERATED (0.08) | 204 TPM (1134 MAX) | 194 | 0.82 | 4.17 |
| *LGR4* | chr11:27405906 | c.666 A>C | p.D222E | NM_018490.2 |  | | disease_causing | Benign (0.109) | TOLERATED (0.6) | 43 TPM (248 MAX) | 45 | 1 | 3.36 |
| *FERMT2* | chr12:53348185 | c.529 T>C | p.S177G | NM_001134999.1 |  | | NA | Benign (0.002) | NA | 26 TPM (310 MAX) | 56 | 1 | 5.92 |
| *IGDCC3* | chr14:65621431 | c.2261 A>C | p.L754R | NM_004884.3 |  | | NA | Probably damaging (0.966) | DAMAGING (0.02) | 10 TPM (89 MAX) | 102 | 0.022 | 2.17 |
| *DIS3L* | chr15:66624307 | c.2630 A>G | p.D877G | NM_001143688.1 |  | | disease_causing | Probably damaging (0.999) | DAMAGING (0.01) | 21 TPM (164 MAX) | 94 | 0.46 | 5.56 |
| *COMMD4* | chr15:75630436 | c.37 G>A | p.D13N | NM_017828.3 |  | | disease_causing | Probably damaging (1) | DAMAGING (0) | 53 TPM (197 MAX) | 23 | 0.998 | 4.33 |
| *SUGP2* | chr15:19130015 | c.1753 T>C | p.R585G | NM_001017392.3 |  | | NA | Benign (0.255) | NA | 227 TPM (818 MAX) | 125 | 0.99 | 3.47 |
| *C21orf91* | chr19:19169325 | c.238 T>C | p.K80E | NM_001100420.1 | 0.000008303 | | polymorphism | Probably damaging (0.961) | TOLERATED (0.31) | 22 TPM (111 MAX) | 56 | 1 | 5.8 |
| *MORC2* | chr21:31332541 | c.1508 G>T | p.T503K | NM_014941.1 |  | | NA | Benign (0.03) | TOLERATED (0.29) | 14 TPM (257 MAX) | 78 | 1 | 6.06 |
| *ELFN2* | chr22:37770170 | c.1405 G>A | p.R469C | NM_052906.3 | 0.00000828 | | disease_causing | Possibly damaging (0.836) | TOLERATED (0.07) | 16 TPM (22 MAX) | 180 | 0.986 | 1.32 |

**Table S4**. List of novel nonsynonymous coding variants segregating within **Family E**. . In light blue are the variants which meet all the filter criteria: 1) novel variants; 2) segregating with the disease; 3) predicted as damaging by at least 2 out of 3 in silico prediction softwares (MUTATION TASTER, POLYPHEN2, SIFT) and 4) highly expressed in the brain and highly conserved (Grantham > 50, PhastCons > 0.4 and GERP >4). The other variants do not meet at least one of the filter criteria. The other variants do not meet at least one of the filter criteria. Chr, chromosome ; Aa, aminoacid; NA, not available.

**References**

1. Milan, G. *et al.* GRN deletion in familial frontotemporal dementia showing association with clinical variability in 3 familial cases. *Neurobiol. Aging* **53**, 193.e9-193.e16 (2017).

2. Coppola, C. *et al.* A cluster of progranulin C157KfsX97 mutations in Southern Italy: clinical characterization and genetic correlations. *Neurobiol. Aging* **49**, 219.e5-219.e13 (2017).

3. Benussi, L. *et al.* A novel deletion in progranulin gene is associated with FTDP-17 and CBS. *Neurobiol. Aging* **29**, 427–435 (2008).

4. Borroni, B. *et al.* Progranulin genetic variations in frontotemporal lobar degeneration: evidence for low mutation frequency in an Italian clinical series. *Neurogenetics* **9**, 197–205 (2008).

5. Borroni, B. *et al.* Founder effect and estimation of the age of the Progranulin Thr272fs mutation in 14 Italian pedigrees with frontotemporal lobar degeneration. *Neurobiol. Aging* **32**, 555.e1–8 (2011).

6. Sassi, C. *et al.* A Novel Splice-Acceptor Site Mutation in GRN (c.709-2 A>T) Causes Frontotemporal Dementia Spectrum in a Large Family from Southern Italy. *J. Alzheimers Dis. JAD* (2016) doi:10.3233/JAD-151170.

7. Gellera, C. *et al.* Ubiquilin 2 mutations in Italian patients with amyotrophic lateral sclerosis and frontotemporal dementia. *J. Neurol. Neurosurg. Psychiatry* **84**, 183–187 (2013).

8. Ratti, A. *et al.* C9ORF72 repeat expansion in a large Italian ALS cohort: evidence of a founder effect. *Neurobiol. Aging* **33**, 2528.e7–14 (2012).

9. Binetti, G. *et al.* Prevalence of TAU mutations in an Italian clinical series of familial frontotemporal patients. *Neurosci. Lett.* **338**, 85–87 (2003).

10. Gidaro, T. *et al.* An Italian family with inclusion-body myopathy and frontotemporal dementia due to mutation in the VCP gene. *Muscle Nerve* **37**, 111–114 (2008).

11. Bersano, A. *et al.* Inclusion body myopathy and frontotemporal dementia caused by a novel VCP mutation. *Neurobiol. Aging* **30**, 752–758 (2009).

12. Viassolo, V. *et al.* Inclusion body myopathy, Paget’s disease of the bone and frontotemporal dementia: recurrence of the VCP R155H mutation in an Italian family and implications for genetic counselling. *Clin. Genet.* **74**, 54–60 (2008).

13. Giovagnoli, A. R. *et al.* Atypical frontotemporal dementia as a new clinical phenotype of Gerstmann-Straussler-Scheinker disease with the PrP-P102L mutation. Description of a previously unreported Italian family. *Neurol. Sci. Off. J. Ital. Neurol. Soc. Ital. Soc. Clin. Neurophysiol.* **29**, 405–410 (2008).

14. Oldoni, E. *et al.* PRNP P39L Variant is a Rare Cause of Frontotemporal Dementia in Italian Population. *J. Alzheimers Dis. JAD* **50**, 353–357 (2016).

15. Le Ber, I. *et al.* Homozygous TREM2 mutation in a family with atypical frontotemporal dementia. *Neurobiol. Aging* **35**, 2419.e23-2419.e25 (2014).

16. Jakovcevski, I., Miljkovic, D., Schachner, M. & Andjus, P. R. Tenascins and inflammation in disorders of the nervous system. *Amino Acids* **44**, 1115–1127 (2013).

17. Dufresne, D. *et al.* Homozygous deletion of Tenascin-R in a patient with intellectual disability. *J. Med. Genet.* **49**, 451–454 (2012).

18. Ayachi, I. El *et al.* Spatiotemporal distribution of tenascin-R in the developing human cerebral cortex parallels neuronal migration. *J. Comp. Neurol.* **519**, 2379–2389 (2011).

19. Hargus, G. *et al.* Tenascin-R promotes neuronal differentiation of embryonic stem cells and recruitment of host-derived neural precursor cells after excitotoxic lesion of the mouse striatum. *Stem Cells Dayt. Ohio* **26**, 1973–1984 (2008).

20. Maruyama, E. *et al.* Brain-derived neurotrophic factor induces cell surface expression of short-form tenascin R complex in hippocampal postsynapses. *Int. J. Biochem. Cell Biol.* **39**, 1930–1942 (2007).

21. Bukalo, O., Schachner, M. & Dityatev, A. Hippocampal metaplasticity induced by deficiency in the extracellular matrix glycoprotein tenascin-R. *J. Neurosci. Off. J. Soc. Neurosci.* **27**, 6019–6028 (2007).

22. Słabicki, M. *et al.* A genome-scale DNA repair RNAi screen identifies SPG48 as a novel gene associated with hereditary spastic paraplegia. *PLoS Biol.* **8**, e1000408 (2010).

23. Schlipf, N. A. *et al.* AP5Z1/SPG48 frequency in autosomal recessive and sporadic spastic paraplegia. *Mol. Genet. Genomic Med.* **2**, 379–382 (2014).

24. Nesbitt, A. *et al.* Exome sequencing expands the mechanism of SOX5-associated intellectual disability: A case presentation with review of sox-related disorders. *Am. J. Med. Genet. A.* (2015) doi:10.1002/ajmg.a.37221.

25. Jones, A. R. *et al.* Stratified gene expression analysis identifies major amyotrophic lateral sclerosis genes. *Neurobiol. Aging* **36**, 2006.e1–9 (2015).


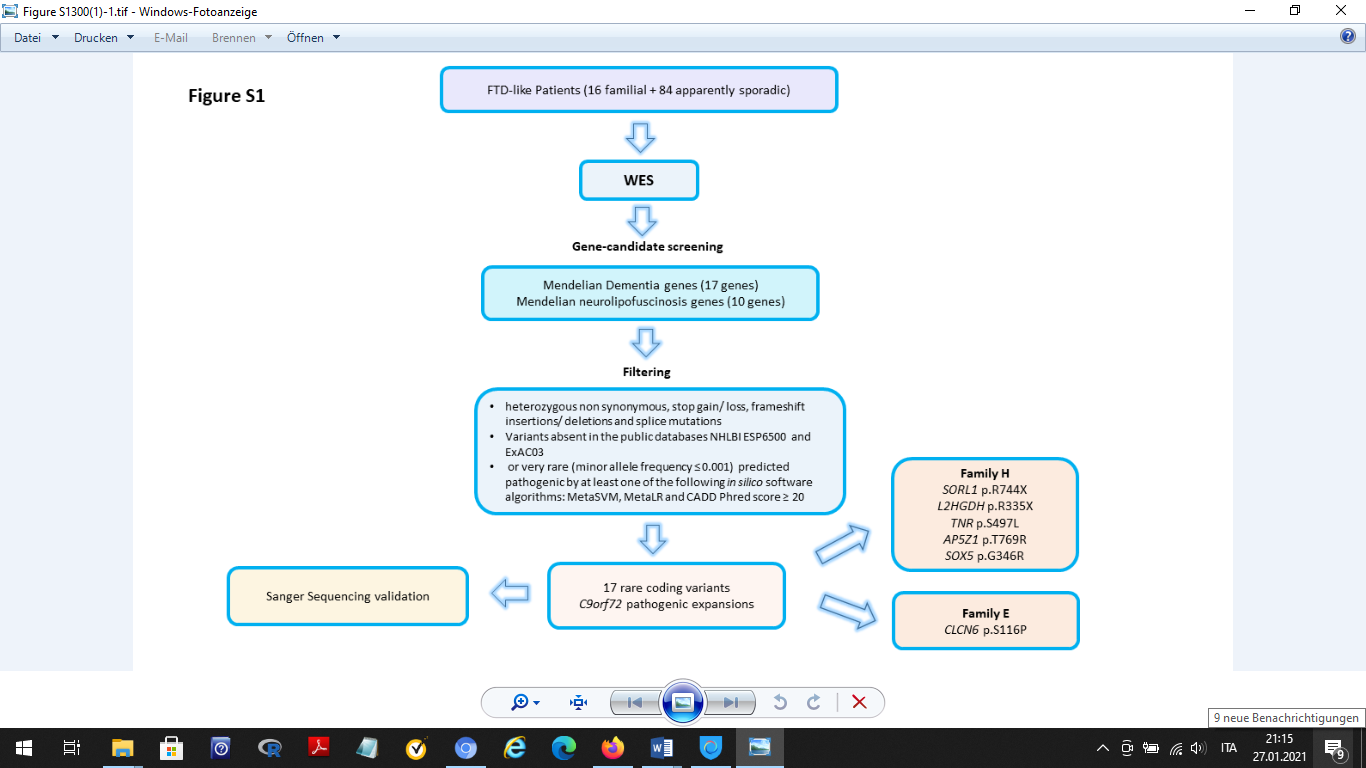


**Figure S1.** Pipeline used in this study. WES, whole generation sequencing.
